# 3D architected temperature-tolerant organohydrogels with ultra-tunable energy absorption

**DOI:** 10.1016/j.isci.2021.102789

**Published:** 2021-06-26

**Authors:** James Utama Surjadi, Yongsen Zhou, Tianyu Wang, Yong Yang, Ji-jung Kai, Yang Lu, Zuankai Wang

**Affiliations:** 1 Department of Mechanical Engineering, City University of Hong Kong, Kowloon, Hong Kong SAR, China; 2 Nano-Manufacturing Laboratory (NML), Shenzhen Research Institute of City University of Hong Kong, Shenzhen 518057, China

**Keywords:** soft matter, mechanical property, metamaterials

## Abstract

The properties of mechanical metamaterials such as strength and energy absorption are often “locked” upon being manufactured. While there have been attempts to achieve tunable mechanical properties, state-of-the-art approaches still cannot achieve high strength/energy absorption with versatile tunability simultaneously. Herein, we fabricate for the first time, 3D architected organohydrogels with specific energy absorption that is readily tunable in an unprecedented range up to 5 × 10^3^ (from 0.0035 to 18.5 J g^−1^) by leveraging on the energy dissipation induced by the synergistic combination of hydrogen bonding and metal coordination. The 3D architected organohydrogels also possess anti-freezing and non-drying properties facilitated by the hydrogen bonding between ethylene glycol and water. In a broader perspective, this work demonstrates a new type of architected metamaterials with the ability to produce a large range of mechanical properties using only a single material system, pushing forward the applications of mechanical metamaterials to broader possibilities.

## Highlights

• The first fabrication of 3D architected organohydrogels by Digital Light Processing
• Two-step toughening effect of organohydrogels by metal coordination and hydrogen bonding
• 3D structures achieved ultra-tunable range of specific energy absorption up to 5000 x
• 3D architected organohydrogels were demonstrated as tunable impact attenuators

## Graphical abstract

**Figure:**
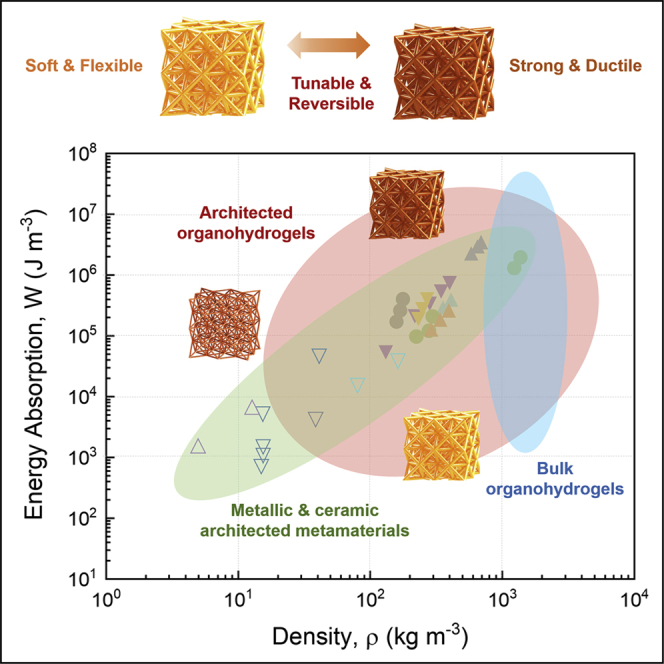

## Introduction

Mechanical metamaterials such as cellular lattices represent a new paradigm of materials due to their unique and unprecedented properties (e.g. ultrahigh specific strength, recoverability, etc.) granted by the incorporation of architectural benefits in addition to the intrinsic properties of its constituents(Surjadi et al., 2019; Zhang et al., 2020b). Typically, the properties of mechanical metamaterials are predetermined by the spatial arrangement of 3D elements, and once manufactured, their mechanical properties remain hard to be tuned or reversibly switched. However, a plethora of engineering applications require metamaterials with tunable mechanical properties (e.g. stiffness, strength) and stress-strain responses (Clough et al., 2019; Koons et al., 2020). For example, protective foams with well-defined architecture and preferential dynamic deformation behavior are crucial to optimally mitigate damage from a wide range of impact loading conditions and prevent injuries (Clough et al., 2019). Pliably tuning the mechanical properties of 3D architected metamaterials is an effective strategy to accommodate for such individualistic requirements.

Previous strategies to bestow mechanical metamaterials with tunable stiffness mainly focus on the development of a core-shell composite or alteration of physical properties via external stimuli. For instance, Jackson et al. incorporated magnetorheological (MR) fluid inside a hollow polymer lattice to achieve tunable stiffness via magnetic field (Jackson et al., 2018). While this approach enables a rapid and sizable change in effective stiffness, only a 35% increase was achieved, which is far below the requirement in practical applications. Similarly, granular particles (GPs) and liquid metal (LM) have also been employed recently as the core of hollow polymeric lattices to achieve wider tunable ranges (Deng et al., 2020; Wang et al., 2019; Zhang et al., 2020a). Another approach featuring significant property changes (stiffness range exceeding 2 orders of magnitude) under elevated temperature was attempted by 3D printing shape memory polymers (SMPs) (Tao et al., 2020; Yang et al., 2019). Despite these recent progresses, it is still challenging to fabricate tunable mechanical metamaterials that exhibit high strength and/or versatile deformability (i.e. toughness/energy absorption) which rivals or exceeds those of non-tunable architected materials(Eckel et al., 2016; Feng et al., 2021; Hernández-Nava et al., 2016; Schaedler et al., 2011; Surjadi et al., 2021a; Zheng et al., 2014, 2016b).

Energy-dissipative materials that can dynamically break and reconstruct molecular interactions offer the potential to resolve the challenge. In particular, tough hydrogels have emerged as a promising choice for applications where high toughness is required (Gong, 2010; Gong et al., 2003). The toughening in tough hydrogel networks is mainly ascribed to non-covalent interactions such as hydrogen bonding (Guo et al., 2014; Hu et al., 2015), electrostatic interaction (Luo et al., 2015; Sun et al., 2013), hydrophobic interaction (Chang et al., 2018;Cui et al., 2019;Fang et al., 2020), host-guest interaction (Liu and Scherman, 2018; Liu et al., 2017), and metal coordination (Sun et al., 2012; Yang et al., 2013). Distinct from covalent interactions, these non-covalent interactions are stimuli-responsive, enabling to dynamically tune the mechanical properties (i.e. toughness, stiffness) of hydrogels via an external stimuli such as temperature (Liang et al., 2019), light (Lee et al., 2018), pH (Liu et al., 2016), and magnetic field (Lee et al., 2019). Among these stimuli, light is preferred owing to its green, remote-controllable, and easy-to-operate properties. For instance, mechanically tunable hydrogels incorporated with Fe^3+^/COO^−^ complex displayed increasing toughness as the concentration of Fe^3+^ increases, while compliant hydrogels are obtained when the Fe^3+^ is reduced to Fe^2+^ in the presence of citric acid and light (Zhang and Silverstein, 2017). This dynamic and light-responsive nature of metal coordination enlightens us to contrive the idea of fabricating metamaterials with tunable mechanical properties (Khare et al., 2021). Nevertheless, hydrogels are easily dehydrated and become brittle under dry or elevated temperature conditions, hindering their applications in many scenarios (Chen et al., 2018).

In this work, we fabricated ultratough 3D architected organohydrogels with tunable energy absorption across 3 orders of magnitude, hinging on the effective incorporation of an energy-dissipative matrix and dynamic metal coordination into 3D printed organohydrogel skeletons. The strengthened hydrogen bonding induced by the binary solvent system (EG and water) further increases toughness and imparts the organohydrogel with enhanced temperature tolerance over conventional hydrogels. Introducing geometry-dependent parameters induced by the incorporation of architecture provides a more versatile platform to manipulate both the physical properties (e.g. density) and mechanical behavior (e.g. stiffness, strength) of organohydrogels. Overall, the creation of 3D architected organohydrogels not only extend the capabilities of hydrogel and organohydrogel-based materials beyond what traditional bulk samples could achieve but also unveils a new route for the manufacture of reconfigurable mechanical metamaterials with an extensive range of tunable toughness for a plethora of engineering applications.

## Results and discussion

### Fabrication and temperature tolerance

**Figure 1:**
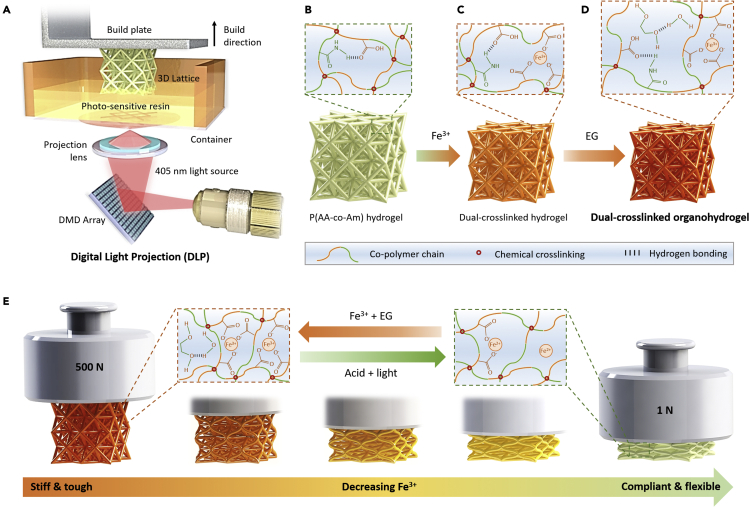
Fabrication of 3D architected dual-crosslinked organohydrogels (A) Schematic illustration of the 3D printing process (DLP) used to fabricate the lattices. (B–D) Illustration showing (B) the hydrogen bonds and covalent crosslinking present in the printed copolymer hydrogel, (C) the formation of metal coordination bond to upon immersing the hydrogel in aqueous Fe^3+^ solution, and (D) the formation of enhanced hydrogen bonds upon immersion of the metal coordinated hydrogel in EG to produce the final dual-crosslinked organohydrogel lattices. (E) Illustration showing the tunability and reversibility of the metal coordination bonds to obtain either ultrahigh toughness or compliant and flexible architected organohydrogels.

Figure 1A shows a schematic illustration of the DLP 3D printing setup used to fabricate the 3D octet lattices in this study. The photosensitive resin mainly consists of acrylic acid (AA) and acrylamide (Am) monomers. The as-printed lattice is therefore composed of p(AA-co-Am) networks (Figure 1B), whose carboxyl (COO^−^) groups form metal coordination bonds with Fe^3+^ ions in aqueous solution (Figure 1C). Followed by the solvent exchange with ethylene glycol (EG), tough 3D architected organohydrogels were ultimately produced (Figure 1D). The metal coordination bonds (Fe^3+^/COO^−^) could be broken down by light-induced reduction (in the presence of citric acid) and reconstructed by suppling with new Fe^3+^ and EG (Figure 1E).

**Figure 2:**
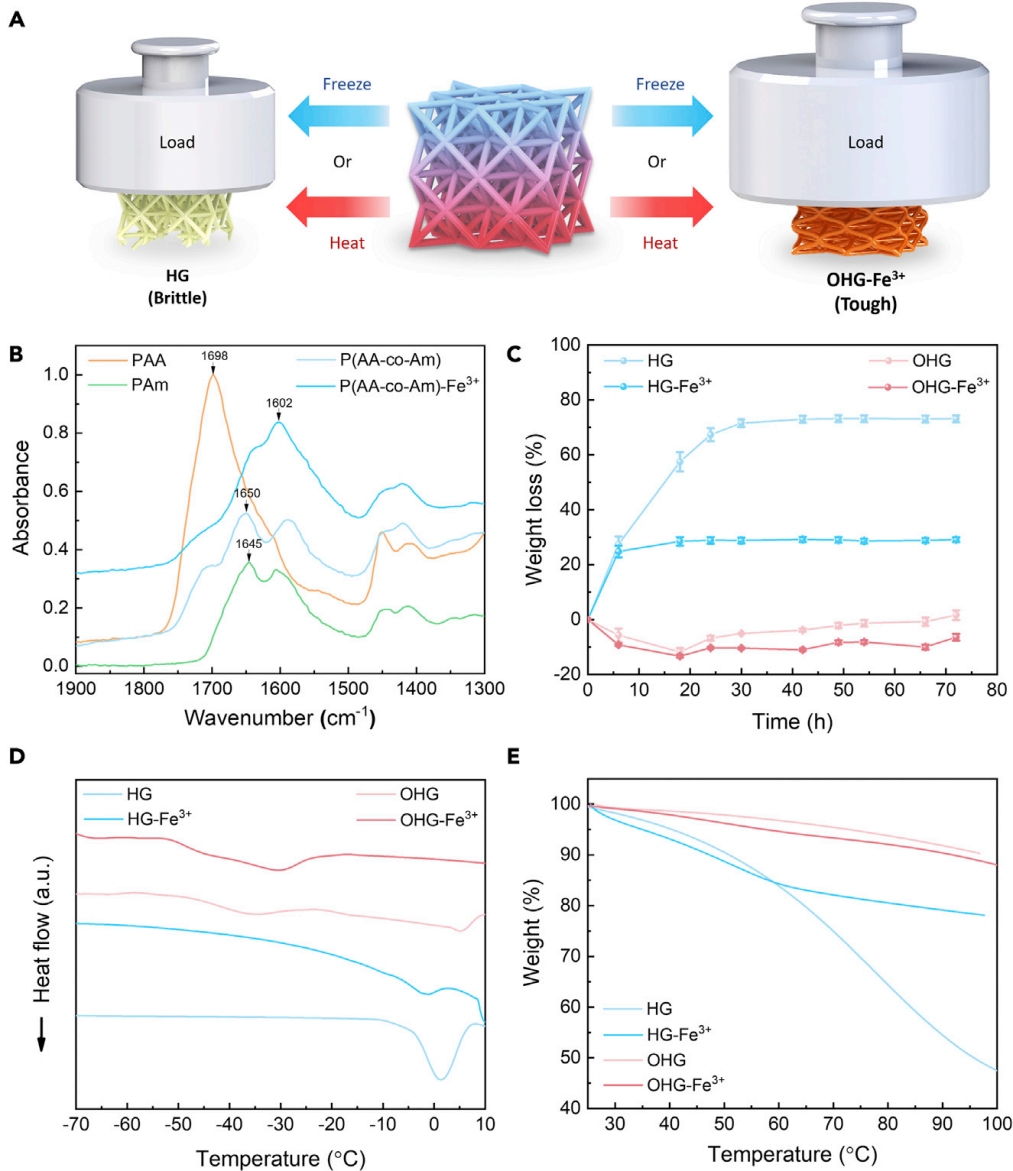
Temperature tolerance of 3D architected dual-crosslinked organohydrogels (A) Schematic illustration showing the mechanical stability of the metal coordinated organohydrogels compared to p(AA-co-AM) hydrogel counterparts across a wide range of temperatures. (B) FTIR spectra showing the peak shifting in the as-printed lattice and subsequent metal coordination. (C) Record of the weight loss versus time for hydrogels (HG), metal coordinated hydrogels (HG-Fe^3+^), organohydrogels (OHG), and metal coordinated organohydrogels (OHG-Fe^3+^). (D) DSC measurements showing the decreased transition/freezing/melting temperatures of the organohydrogels (OHG and OHG-Fe^3+^) compared to the hydrogels (HG and HG-Fe^3+^). (E) TGA results showing the significantly reduced weight loss of the organohydrogels in comparison to hydrogels at elevated temperatures.

Figure 2A illustrates the distinctive temperature tolerance between the fabricated 3D hydrogel (HG) and organohydrogel (OHG) with metal coordination bonds. The HG lattice can be easily frozen (sub-zero temperature) and dehydrated (above room temperature), making it intrinsically brittle when subjected to extreme temperatures. On the other hand, the OHG lattice can retain its toughness within a significantly larger temperature range. The composition of the 3D printed lattice was analyzed by Fourier-transform infrared (FTIR) spectroscopy in Figure 2B. The strong peak at 1698 cm^−1^ (carboxylic acid group of polyacrylic acid) and the peak at 1645 cm^−1^ (amide group of polyacrylamide) were shifted to 1650 cm^−1^ in p(AA-co-Am), indicating the hydrogen bonding between COO^−^ and NH_2_ groups (Figure 1B). After metal coordination with Fe^3+^, the peak at 1650 cm^−1^ was shifted to 1602 cm^−1^. Weight loss of HG and OHG lattices with time was recorded under ambient conditions (22 ± 3⁰C, 50% ± 10RH). While the hydrogels rapidly lost weight due to dehydration (fully dehydrated after ~30 hr as shown by the constant weight), the organohydrogels exhibited negligible weight loss even after 3 days (Figures 2C and S1), indicating the non-drying properties of OHG lattices.

The temperature tolerance of the 3D (organo)hydrogels was further investigated via differential scanning calorimetry (DSC), thermogravimetric analysis (TGA), and dynamic mechanical analysis (DMA). A transition peak corresponding to the formation of ice crystals was observed at approximately 0⁰C for the hydrogels (i.e. HG and HG-Fe^3+^), while this peak was shifted to about - 40⁰C for the organohydrogels (Figure 2D), implying that the binary solvent provides a significant enhancement to the anti-freezing properties. Similarly, the temperature tolerance of the (organo)hydrogels under elevated temperature was further accentuated via TGA (Figure 2E). While the organohydrogels (OHG and OHG-Fe^3+^) retained 90% of the original weight even when heated to 100⁰C, the hydrogels experienced a substantial weight loss when heated to the same temperature (more than 50% and 20% loss for the HG and HG-Fe^3+^, respectively). The mechanical stability of the (organo)hydrogels under temperature change was verified via DMA (Figure S2). The hydrogels exhibited a drastic increase in storage modulus around 0⁰C due to the freezing of the “free” water molecules inside. Conversely, the organohydrogels maintained a stable storage modulus down to −40⁰C, which is in good agreement with the DSC results. Overall, these results demonstrate the enhanced temperature tolerance of the organohydrogel compared to the hydrogel in retaining flexibility and/or toughness.

### Mechanical characterization and tunability

**Figure 3:**
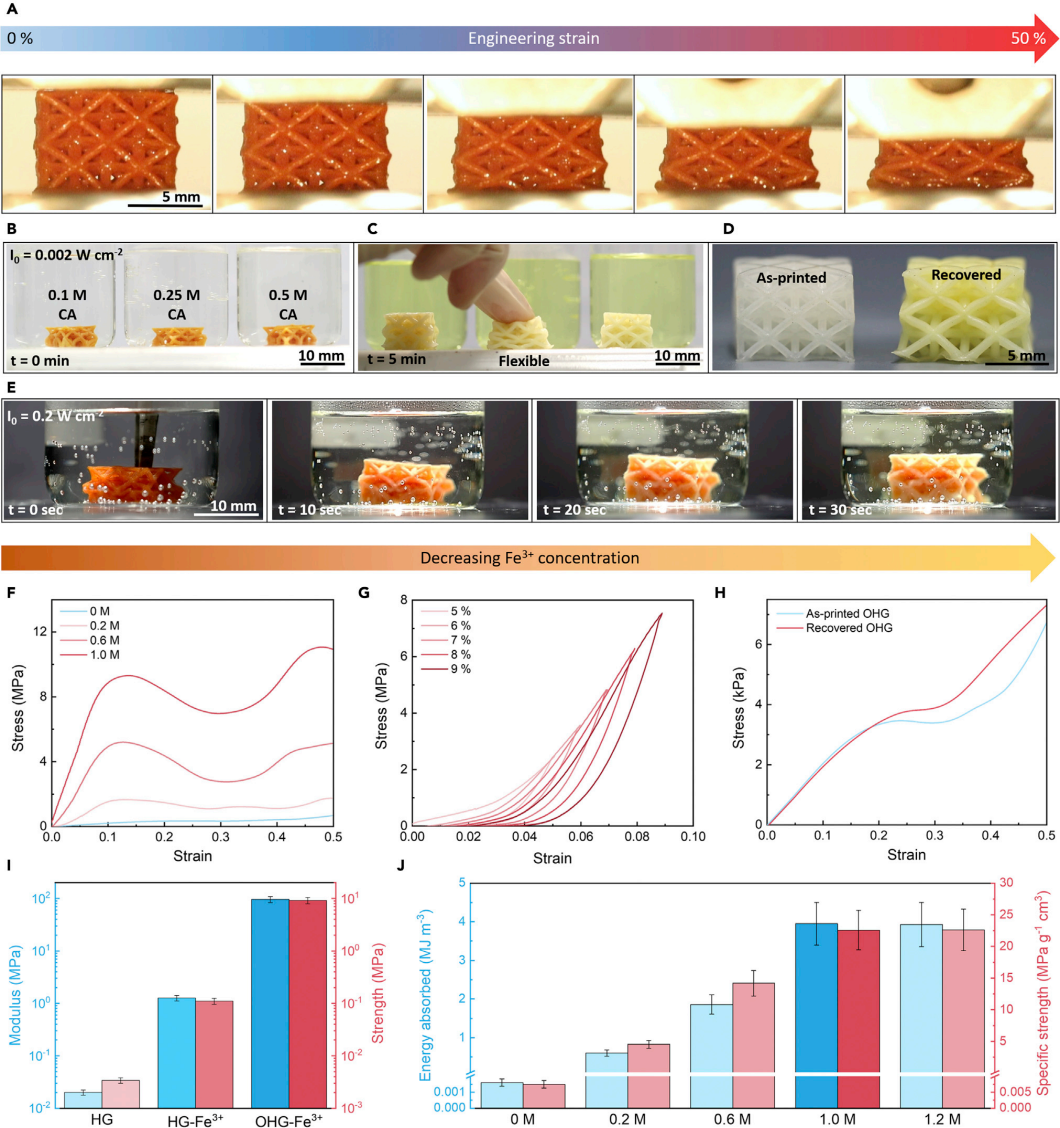
Mechanical characterization of 3D architected lattices with tunable mechanical properties (A) *In situ* deformation of the dual-crosslinked octet lattices under uniaxial compression. (B) Immersion of the deformed lattices under various concentrations of citric acid (CA) at constant light intensity of 0.002 W cm^−2^. (C and D) Showing the regained shape and flexibility of the recovered lattices upon immersion in CA under light. (E) Showing the accelerated recovery of the deformed lattices under higher light intensity (0.2 W cm^−2^) immersed in 0.25 M of CA. (F) Stress-strain curves obtained from the uniaxial compression of the dual-crosslinked organohydrogel lattices immersed in different concentrations of Fe^3+^ solution. (G) Loading-unloading curves of the dual-crosslinked lattices. (H) Comparison between the mechanical properties of the as-fabricated organohydrogel with recovered organohydrogel, demonstrating the reversibility of the metal coordination bonds. (I) Young's modulus and compressive strength comparison between the lattices obtained at each fabrication step (from HG to HG-Fe^3+^, and finally OHG-Fe^3+^). (J) Comparison of the energy absorption capabilities and specific strength between the dual-crosslinked organohydrogels immersed in different concentrations of Fe^3+^ solutions.

Figure 3A and Video S1 shows the *in situ* deformation of the 3D organohydrogel with metal coordination bonds (OHG-Fe^3+^) under uniaxial compression. The architected OHG-Fe^3+^ exhibited a gradual layer-by-layer buckling of its struts without any apparent fracture, which is a typical behavior of ductile/deformable lattices. Our intuition prompted us to recover the buckled lattices by immersing them in the citric acid solution in the presence of light where the Fe^3+^ was reduced to Fe^2+^ and the EG was drained up. The complete geometrical recovery was clearly observed (Figure 3C and Video S2). The time taken for the geometrical recovery, independent of the citric acid concentration (Figure 3B), was drastically reduced from 5 min to 30 s when the light intensity (Io) was increased from ~0.002 W cm^−2^ to ~0.2 W cm^−2^ (Figure 3E). Figure S3B summarizes the time taken for the compressed OHG-Fe^3+^ lattice to recover to its original size at various citric acid concentrations and light intensities. The mechanical recovery of the compliant and flexible lattice is verified by the compression test (Figure 3H). The geometrical and mechanical recovery indicates that the tough 3D lattices can be readily softened. Reprocessing the softened 3D lattices can reproduce the tough lattices, as indicated in Figure S3A, by the resupplementation of Fe^3+^ and EG.

Video S1. *In situ* uniaxial compression of OHG-Fe3+ lattices up to 50% strain (50x speed)

Video S2. Recovery of deformed OHG-Fe3+ lattices via deconstruction of metal coordination bonds under light, Io ~ 0.2 W cm-2 (3x speed)

The effect of Fe^3+^ concentration on the mechanical properties was quantitatively investigated (Figure 3F). As the concentration of Fe^3+^ increases, the compressive Young's modulus (E) and strength (σ_y_) of the octet organohydrogel lattices continually increases (from E ~ 0.02 MPa and σ_y_ ~ 0.003 MPa at 0 M) until it plateaus at 1.0 M (E ~ 98.2 MPa and σ_y_ ~ 9.5 MPa). This amounts to ~490,000% and ~320,000% difference in modulus and strength respectively, which has not yet been achieved for mechanical metamaterials. A series of loading-unloading experiments were also performed to determine the elastic limit and recovery of the tough architected organohydrogels (Figure 3G). The organohydrogel lattices demonstrated near 100% recovery under 10% strain, which is the strain at which most brittle materials fracture.

To quantify the contribution of each fabrication step (immersion in aqueous Fe^3+^ solution and EG, as shown in Figure 1B) to the toughening of the architected organohydrogels, the mechanical properties of the hydrogels without (HG) and with (HG-Fe^3+^) metal coordination bonds were compared against the organohydrogel lattices with metal coordination bonds (OHG-Fe^3+^), as shown in Figure 3I. The concentration of Fe^3+^ used was 1.0 M in this case. It was discovered that the octet lattices exhibited a two-stage strengthening behavior. A significant increase in modulus (~6300%) and strength (~3700%) was observed upon immersion in Fe^3+^ solution in the first stage, and another drastic increase in modulus (~7700%) and strength (~8600%) was achieved upon immersion in EG in the second stage.

Energy absorption capability is a highly desired crucial parameter for engineering applications as it demonstrates both the strength and deformability of a material, whereas specific strength (strength/density) provides the load bearing capability of a cellular material with respect to its weight. The energy absorption per unit volume and specific strength of the 3D architected organohydrogels with different Fe^3+^ concentrations are provided in Figure 3J. The tunable ranges in toughness and specific strength were calculated to be ~350,000% and ~400,000%, respectively. Overall, the inclusion of Fe^3+^ and EG results in ultratough 3D organohydrogels with an unprecedented tunability and reproducibility.

### Origins of temperature tolerance

High temperature tolerance (i.e. anti-freezing and non-drying properties) of a material is highly desired for a broad range of applications. For decades, sustaining a long-term stability under extreme temperature conditions has been the Achilles heel for hydrogels in many applications (Chen et al., 2018). Various approaches have been attempted to fabricate anti-freezing and non-drying hydrogels, including using high concentration of salt (calcium chloride) (Morelle et al., 2018), ionic liquid (Ding et al., 2017; Ren et al., 2019), binary solvent consisting of water and polyol (i.e. EG, glycerol) (Lou et al., 2019; Xia et al., 2019), and zwitterionic osmolytes (Sui et al., 2020). In this work, an EG/water binary solvent was used to improve the temperature tolerance of the hydrogel lattices. EG is known to be an effective cryoprotectant owing to its tendency to form hydrogen bonding with water, disrupting the hydrogen bonds (H-bonds) between water molecules when the temperature is lower than the crystallization temperature of water (Lou et al., 2019; Mo et al., 2019). As expected, the OHG-Fe^3+^ lattice demonstrated excellent anti-freezing and non-drying properties (Figures 2, S1, and S2).

### Origins of ultra-tunable toughness

To achieve exceptional toughness in either hydrogels or organohydrogels, an energy-dissipative matrix is indispensable. Here, we judiciously incorporated the metal coordination motif into the OHG lattices, which not only contributed to the enhancement of toughness but also the reversibility of toughness. The elevated toughness stems from the non-covalent interactions exhibited in the OHG lattices, namely the hydrogen bonding (between carboxyl group and amide group) and metal coordination (between carboxyl group and Fe^3+^) as shown in Figures 1B–1D. The reversibility of toughness is mainly attributed to the dynamic COO^−^/Fe^3+^ coordination. When the Fe^3+^ ions in the OHG lattice are reduced to Fe^2+^ ions in the presence of citric acid and light, the metal coordination is greatly weakened, resulting in reduced toughness(Peng et al., 2008, 2017; Zhang and Silverstein, 2017). When supplied with new Fe^3+^ ions, the metal coordination can be regained. The toughness of the OHG-Fe^3+^ lattices, therefore, can be reversibly tuned.

The considerable increase in modulus and strength from HG to HG-Fe^3+^ is caused by the formation of metal coordination bond between Fe^3+^ and -COO^-^ in polyacrylic acid, which is also responsible for the tunable mechanical properties in the 3D lattices (Figures 1C and 2B).(Lin et al., 2015) Among the multi-valent cations which can form coordinated complexes with COO^−^, Fe^3+^ is known to exhibit one of the strongest bonds owing to the trivalent ionic interaction(Zheng et al., 2016a). The formation of metal coordination bonds also resulted in a volumetric shrinkage, ascribed to the chain confinement caused by the strong interfacial interaction(Shao et al., 2017; Zhang and Silverstein, 2017). This produced a denser crosslinked polymer network, which leads to a further enhancement in mechanical strength of the dual-crosslinked architected hydrogel (Figure S4). Meanwhile, the gradual strengthening of the lattices as the concentration of Fe^3+^ increases is due to the increasing number of ionic crosslinks, while the plateau after 1.0M is caused by the absence of vacant carboxyl groups for the excess Fe^3+^ to bond with at higher concentrations.

In addition to the enhancement arising from the metal coordination, subsequent immersion of the dual-crosslinked hydrogel in EG (from HG-Fe^3+^ to OHG-Fe^3+^) induced another drastic increase in mechanical properties (i.e. stiffness, strength, toughness). This is presumably caused by the reduced solvation of water to the copolymer networks and increased H-bonds (between water and EG) in the OHG-Fe^3+^ lattice when EG is added. The water molecules can hydrate the polymer chains, especially when a hygroscopic polymer (polyacrylic acid, in our case) is the main constituent, via the solvation effect. However, this solvation effect is largely weakened when EG is introduced, forming a binary solvent system where stronger EG-water interaction is predominant over the EG-EG and water-water interactions (Chen et al., 2013; Kumar et al., 2012). The EG-water mixture could also enhance the H-bonds between solvent and the polymer network. In the binary solvent, the presence of water molecules could serve as a bridge between the carbonyl groups of the polymers and hydroxyl groups of EG, providing new interaction sites for strong hydrogen bonding which increases the binding energies in the system(Han et al., 2018). Therefore, the increased non-covalent interactions (metal coordination bonds and H-bonds) and reduced solvation resulted in the synergistic two-stage enhancement of mechanical properties.

Furthermore, the shape of the deformed lattices could be recovered to its uncompressed state upon exposure to light in the aqueous citric acid solution. As mentioned above, Fe^3+^ ions can be reduced to Fe^2+^ ions when irradiated by light in the presence of citric acid. This change in the oxidation state of the Fe ions was indicated by the color change of the lattices (Figures 3C and 3D). As the orange color of the deformed lattice faded away, it began to absorb water and swell. The breakdown of coordination bonds and the subsequent swelling were responsible for the shape recovery of the lattices. Although some of the produced Fe^2+^ could also bind to the COO^−^ groups, these bonds are significantly weaker than the trivalent ionic interaction between the Fe^3+^ and COO^−^. By washing the sample several times in water, the color eventually faded away and EG was drained up, restoring the mechanical properties of the lattices to its original, compliant state (Figure 3H). The notable decrease in recovery time as the light intensity increases is attributed to the increase in energy provided to weaken the metal coordination bonds (Figure 3E).

### Comparison with previous works

**Figure 4:**
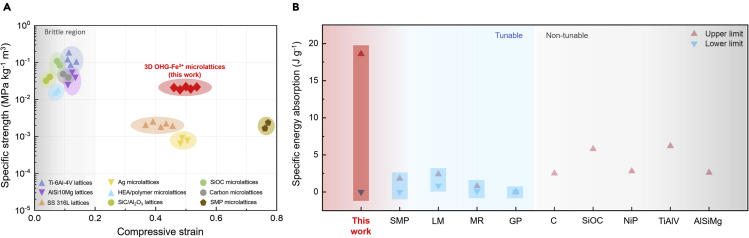
Mechanical property comparison with previously reported lattices/microlattices (A) Specific strength versus compressive strain comparison between our 3D architected dual-crosslinked organohydrogels (3D OHG-Fe^3+^) with other reported lattices, demonstrating its combination of high specific strength and deformability. The compressive strain was taken as the strain at which fracture occurs or the strain at which the lattices were compressed to. (B) Specific energy absorption (SEA) comparison between our 3D OHG-Fe^3+^ compared to previously reported lattices, showing that our work could exhibit superior toughness despite demonstrating the widest range in tunable toughness. The referenced data was extracted from the following: Ti-6Al-4V (TiAlV) (Hernández-Nava et al., 2016), AlSi10Mg (AlSiMg) (Yan et al., 2014), SS 316L (Tsopanos et al., 2010), Ag (Saleh et al., 2017), NiP (Zheng et al., 2016b), HEA/polymer (Surjadi et al., 2018), SiC/Al_2_O_3_ (Mei et al., 2019), SiOC (Eckel et al., 2016), Carbon (C) (Jacobsen et al., 2011), shape memory polymer (SMP) (Yang et al., 2019), liquid metal (LM) (Deng et al., 2020), magnetorheological fluid (MR) (Jackson et al., 2018), and granular particles (GPs) (Wang et al., 2019).

We compared the mechanical performances of our 3D architected dual-crosslinked organohydrogels with previously reported lattices in terms of specific strength, compressive strain, and specific energy absorption (Figure 4).(Eckel et al., 2016; Hernández-Nava et al., 2016; Jacobsen et al., 2011; Mei et al., 2019; Saleh et al., 2017; Schaedler et al., 2011; Surjadi et al., 2018, 2021b; Tsopanos et al., 2010; Yan et al., 2014; Yang et al., 2019; Zheng et al., 2014, 2016b) From Figure 4A, it could be seen that the OHG-Fe^3+^ lattices possess high specific strength, outperforming or rivaling that of other lattices. For instance, the OHG-Fe^3+^ outperforms silver microlattices (Ag), SMP microlattices, and even stainless-steel lattices (SS 316L) in terms of specific strength while maintaining deformability. Other lattices with higher specific strength, such as the Ti-6Al-4V lattices and SiOC microlattices, typically exhibit brittle or catastrophic failure upon mechanical loading exceeding its fracture stress. Consequently, the OHG-Fe^3+^ lattices surpass most reported lattices in terms of specific toughness (i.e. specific energy absorption) (Figure 4B). High specific energy absorption is a critical parameter for engineering applications as it highlights the ability of a material to absorb energy efficiently (i.e. requiring less material), enabling the creation of lightweight energy absorbers. Coupled with an unsurpassed range for tunable mechanical properties, our 3D architected organohydrogels provides immense versatility for engineering applications.

### Tunable impact attenuation

**Figure 5:**
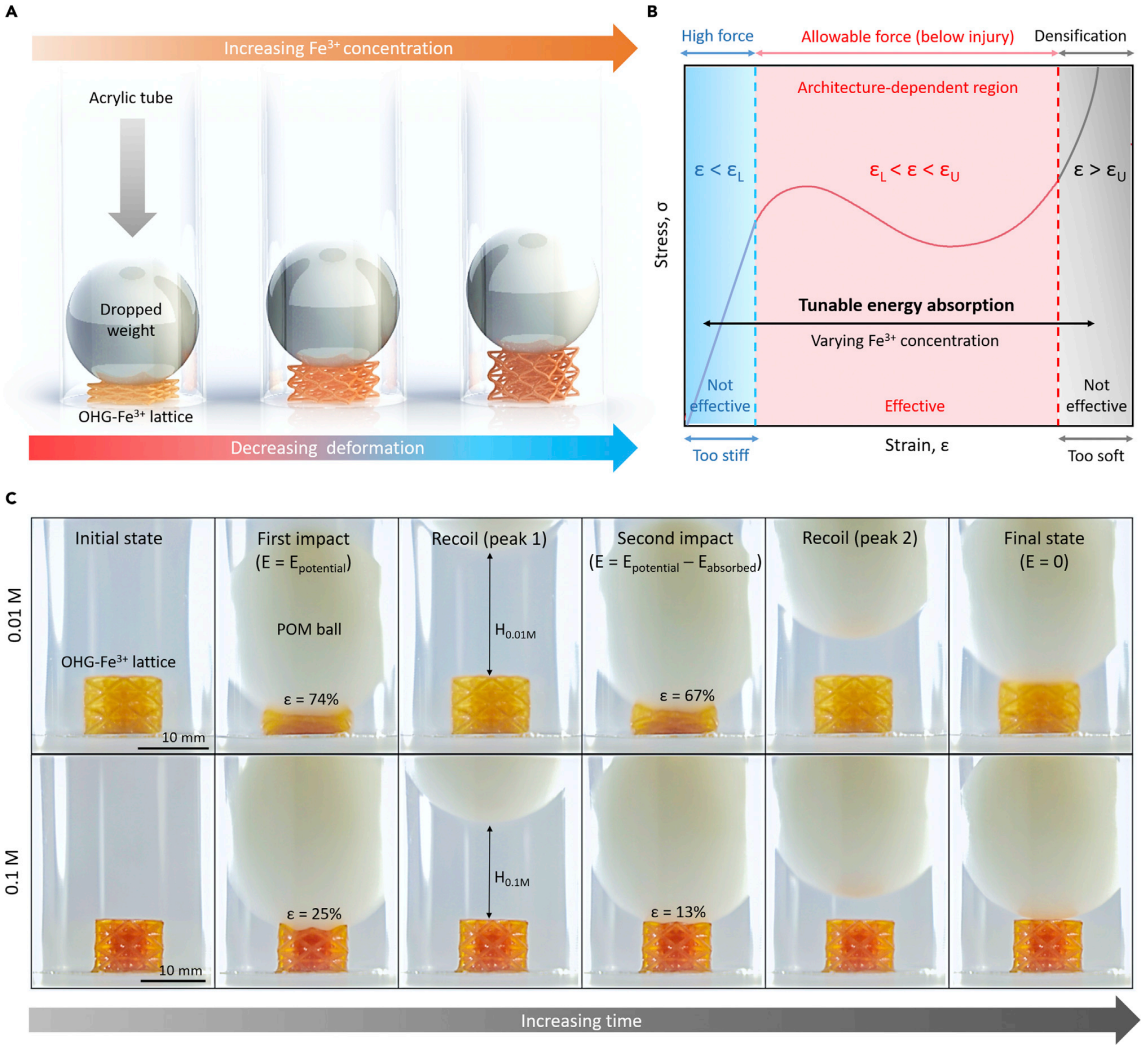
3D architected dual-crosslinked organohydrogels as tunable impact attenuators (A) Illustration showing the concept of tunable impact mitigation using the 3D OHG-Fe^3+^ lattices. (B) Typical stress-strain response of the 3D OHG-Fe^3+^ lattices, which could be tailored to suit for a wide range of impact forces. (C) Ball drop tests on the 3D OHG-Fe^3+^ lattices with varying Fe^3+^ concentration, demonstrating the tunable deformation capability of our lattices to control the transmitted peak impact force. E represents the impact energy, while E_potential_ represents the initial potential energy of the dropped ball, and E_absorbed_ is the energy absorbed by the lattice upon impact.

Facilitated by the ultra-tunable energy absorption of our 3D architected organohydrogels, we attempted to demonstrate its potential application in personal protective equipment (PPE). Unlike conventional protective foams with randomly arranged internal pores and a fixed stress-strain response, the peak impact force and deformation behavior of our 3D architected organohydrogels could be tailored to be below the injury criterion for a wide range of impact scenarios. This is because the inclusion of architecture means that the properties and deformation behaviors of the organohydrogel are not only governed by material-dependent parameters such as degree of crosslinking and relative ratio of the different components but are also controlled by geometrical-dependent parameters such as the type of architecture, relative density, and spatial arrangement of its 3D features. Specifically, the feasibility of our 3D lattice as an impact attenuator (IA) was verified by a simple ball-dropping test (Figure 5). Figure 5A illustrates the concept of tunable IA with our architected organohydrogels. Higher concentration of Fe^3+^ results in smaller deformation during impact, at the expense of increased transmitted force. Note that an architected organohydrogel that is too soft (low concentration of Fe^3+^) or too stiff (high concentration of Fe^3+^) would not be practical as IAs, as it would result in large transmitted force. The tunability of our architected organohydrogels allows their stress-strain response to be tailored to provide the optimal IA performance for an extensive range of impact scenarios by varying the Fe^3+^ concentration. The deformation could therefore be controlled to lie within the effective range, ε_L_ < ε < ε_U_, where ε_L_ represents the lower strain limit such that the transmitted force is below the injury criterion (Greenwald et al., 2008; Gurdjian et al., 1966), while ε_U_ is the upper strain limit before densification starts to occur (Figure 5B). Upon densification, additional deformation is achieved at the cost of increased force transmission, leaving the excess impact energy to be absorbed by the person the IA was supposed to protect (Clough et al., 2019). Here, ε_L_ is mainly dependent on the magnitude of impact energy that the IA is subjected to, which could vary significantly for different impact situations, while ε_U_ is controlled by the architecture and relative density of the lattices. As an example, dropping a plastic ball (polyoxymethylene, POM, ~ 45 g) onto OHG-Fe^3+^ lattices with different Fe^3+^ concentration, guided by a 50 cm high acrylic tube, results in different deformation (Figure 5C). The OHG-Fe^3+^ lattice with lower Fe^3+^ concentration (0.01 M) was deformed beyond its densification strain (ε > 60%) even after the second bounce, implying that the lattice was too soft to sufficiently absorb the impact energy (Video S3). Conversely, the OHG-Fe^3+^ lattice immersed in higher Fe^3+^ concentration (0.1 M) only exhibited ε ~ 25% upon the first impact, signifying that it is sufficiently stiff to absorb the impact energy (Video S4). Further increasing the Fe^3+^ concentration would experience reduced deformation, and the OHG-Fe^3+^ lattices can continue to effectively function as an IA till the deformation falls out of the effective range (i.e. ε < ε_L_). A generalization of the various factors which influence the mechanical properties of the architected organohydrogel for engineering applications, as well as a numerical model for mechanical property analysis are discussed in more detail in supplemental information S1 and S2.

Video S3. Ball drop test on OHG-Fe3+ lattices immersed in 0.01 M Fe3+ solution (0.1x speed)

Video S4. Ball drop test on OHG-Fe3+ lattices immersed in 0.1 M Fe3+ solution (0.1x speed)

In summary, we demonstrated the fabrication of novel metamaterials with simultaneous high energy absorption and ultrawide tunability by the effective combination of hydrogen bonding and dynamic metal coordination. This method not only extends the capabilities of organohydrogels beyond traditional bulk samples through the incorporation of architecture but also bridges the gap between high energy absorption and versatile tunability in metamaterials. Moreover, the EG and water binary solvent system gives rise to enhanced temperature tolerance over conventional hydrogels. We further demonstrated the application of our architected organohydrogels as tunable impact attenuators. This strategy could be applied in the creation of next generation adaptable mechanical metamaterials for a variety of structural and functional applications.

### Limitations of the study

In this study, the toughening of the 3D architected organohydrogels is ascribed to the formation of metal coordination and reconfiguration of hydrogen bonding due to the introduction of EG. However, the microstructural evolution in each fabrication step cannot be observed directly by scanning electron microscopy as it requires the complete removal of solvent in the samples. Therefore, Fourier transform infrared spectroscopy (FTIR) remains the only technique to characterize the changes in chemical bonds and molecular interactions within the hydrogel/organohydrogel samples.

## Supporting citations

Ashby, 2006; Deshpande et al., 2001a; Deshpande et al., 2001b; Dong et al., 2015; Fleck et al., 2010; Greer and Deshpande, 2019; He et al., 2017; Jiang and Wang, 2016; Kudo et al., 2019; Lifson, 2019; Maskery et al., 2016; O’Masta et al., 2017; Rong et al., 2017; Schaedler and Carter, 2016; Schaedler et al., 2014; Schwaiger et al., 2019; Tancogne-Dejean et al., 2016; Thiyagasundaram et al., 2010; Warmuth et al., 2016.

## STAR★METHODS

### Key resources table

**Table:** 

REAGENT or RESOURCE	SOURCE	IDENTIFIER
**Chemicals, peptides, and recombinant proteins**		

Acrylamide	Sigma-Aldrich	Cat#A8887; CAS: 79-06-1
Acrylic acid	Sigma-Aldrich	Cat#8.00181; CAS: 79-10-7
N,N'-methylenebisacrylamide	Sigma-Aldrich	Cat#146072; CAS: 110-26-9
Phenylbis(2,4,6-trimethylbenzoyl)phosphine oxide	Sigma-Aldrich	Cat#511447; CAS: 162881-26-7
Dimethyl sulfoxide	Dieckmann (Hong Kong) Chemical Industry	Cat#D103272; CAS: 67-68-5
Iron(III) chloride hexahydrate	Sigma-Aldrich	Cat#236489; CAS: 10025-77-1
Ethylene glycol	Sigma-Aldrich	Cat#102466; CAS: 107-21-1

**Software and algorithms**		

SolidWorks	Dassault Systèmes	https://www.solidworks.com/

### Resource availability

#### Lead contact

Further information and requests for resources and reagents should be directed to and will be fulfilled by the lead contact, Zuankai Wang (zuanwang@cityu.edu.hk).

#### Materials availability

The study did not generate any unique reagents.

#### Data and code availability

• All data reported in this paper will be shared by the lead contact upon request.
• This paper does not report original code.
• Any additional information required to reanalyze the data reported in this paper is available from the lead contact upon request.

### Method details

#### Fabrication of architected organohydrogels

The precursor photosensitive resin consists of 0.25 mmol of acrylamide (Am) and 0.025 mmol of acrylic acid (AA) monomers, 0.03 mmol of N,N'-methylenebisacrylamide (MBAA) crosslinker, and 0.05 mmol of phenylbis(2,4,6-trimethylbenzoyl)phosphine oxide (BAPO) photoinitiator, which were all well dissolved in the solvent of 20 g dimethyl sulfoxide (DMSO). The stretching-dominated octet lattices were designed using a CAD software (SolidWorks) and fabricated using a Digital Light Projection (DLP) 3D printer (Micromake L3+) with 405 nm light source. The slicing distance used was 50 μm, and the curing time for each 2D layer was set at 10 s. The geometries of the copolymer lattices were measured to be approximately 10.0 mm x 10.0 mm x 10.0 mm. After printing, the fabricated lattices were then washed in water, followed by immersion in aqueous FeCl_3_ solution for 12 hours, then ethylene glycol (EG) for 5 hours to ultimately produce the metal coordinated architected organohydrogels.

#### Materials characterization

Fourier-transform infrared (FTIR, PE Spectrum One) measurement were conducted to determine the presence of functional groups in the fabricated lattices. Thermogravimetric Analysis and Differential Scanning Calorimetry measurements (TG/DSC, METTLER TOLEDO, TGA/DSC 3+) were employed to evaluate its transition temperatures and weight loss under elevated temperatures, respectively. TGA and DSC experiments were conducted under Argon atmosphere using a heating rate of 5°C/min. Dynamic mechanical analysis (DMA, METTLER TOLEDO, DMA 1) were performed to investigate the mechanical stability of the lattices at various temperatures. A heating rate of 5°C/min, displacement of 20 μm, and frequency of 5 Hz was used for all the DMA experiments. The weight losses of the hydrogels and organohydrogels at ambient conditions (i.e. 22°C ± 3, 50% ± 10 RH) were recorded using an analytical balance (± 0.0001 g).

#### Mechanical testing and characterization

The experimental setup for the in situ uniaxial compression tests were conducted at room temperature on the MTS RT/30 Electro-Mechanical Material Testing System controlled by TestWorks 4.0 software. A high-speed video camera (Canon™ EOS-1D X Mark II) equipped with a telephoto macro lens (Canon™ EF 100–400 mm f/4.5–5.6 L IS II USM Lens with Canon™ 77 mm 500D close-up lens attachment) was used to observe the deformation behavior and recovery of the lattices during compression and ball impact testing. Uniaxial compression tests were performed on the fabricated lattices at a prescribed strain rate of 10^-3^ s^-1^.

## Footnotes

Supplemental information can be found online at https://doi.org/10.1016/j.isci.2021.102789.

## References

[bib1] Ashby M.F. (2006). The properties of foams and lattices. Philos. Trans. Math. Phys. Eng. Sci..

[bib2] Chang X., Geng Y., Cao H., Zhou J., Tian Y., Shan G., Bao Y., Wu Z.L., Pan P. (2018). Dual-crosslink physical hydrogels with high toughness based on synergistic hydrogen bonding and hydrophobic interactions. Macromolecular Rapid Commun..

[bib3] Chen F., Zhou D., Wang J., Li T., Zhou X., Gan T., Handschuh-Wang S., Zhou X. (2018). Rational fabrication of anti-freezing, non-drying tough organohydrogels by one-pot solvent displacement. Angew. Chem..

[bib4] Chen Y., Ozaki Y., Czarnecki M.A. (2013). Molecular structure and hydrogen bonding in pure liquid ethylene glycol and ethylene glycol–water mixtures studied using NIR spectroscopy. Phys. Chem. Chem. Phys..

[bib5] Clough E.C., Plaisted T.A., Eckel Z.C., Cante K., Hundley J.M., Schaedler T.A. (2019). Elastomeric microlattice impact attenuators. Matter.

[bib6] Cui X.F., Zheng W.J., Zou W., Liu X.Y., Yang H., Yan J., Gao Y. (2019). Water-retaining, tough and self-healing hydrogels and their uses as fire-resistant materials. Polym. Chem..

[bib7] Deng F., Nguyen Q.-K., Zhang P. (2020). Multifunctional liquid metal lattice materials through hybrid design and manufacturing. Additive Manufacturing.

[bib8] Deshpande V., Ashby M., Fleck N. (2001). Foam topology: bending versus stretching dominated architectures. Acta Materialia.

[bib9] Deshpande V.S., Fleck N.A., Ashby M.F. (2001). Effective properties of the octet-truss lattice material. J. Mech. Phys. Sol..

[bib10] Ding Y., Zhang J., Chang L., Zhang X., Liu H., Jiang L. (2017). Preparation of high-performance ionogels with excellent transparency, good mechanical strength, and high conductivity. Adv. Mater..

[bib11] Dong L., Deshpande V., Wadley H. (2015). Mechanical response of Ti–6Al–4V octet-truss lattice structures. Int. J. Sol. Structures.

[bib12] Eckel Z.C., Zhou C., Martin J.H., Jacobsen A.J., Carter W.B., Schaedler T.A. (2016). Additive manufacturing of polymer-derived ceramics. Science.

[bib13] Fang X., Li Y., Li X., Liu W., Yu X., Yan F., Sun J. (2020). Dynamic hydrophobic domains enable the fabrication of mechanically robust and highly elastic poly (vinyl alcohol)-based hydrogels with excellent self-healing ability. ACS Mater. Lett..

[bib14] Feng X., Surjadi J.U., Fan R., Li X., Zhou W., Zhao S., Lu Y. (2021). Microalloyed medium-entropy alloy (MEA) composite nanolattices with ultrahigh toughness and cyclability. Mater. Today.

[bib15] Fleck N.A., Deshpande V.S., Ashby M.F. (2010). Micro-architectured materials: past, present and future. Proc. R. Soc. A.

[bib16] Gong J.P. (2010). Why are double network hydrogels so tough?. Soft Matter.

[bib17] Gong J.P., Katsuyama Y., Kurokawa T., Osada Y. (2003). Double-network hydrogels with extremely high mechanical strength. Adv. Mater..

[bib18] Greenwald R.M., Gwin J.T., Chu J.J., Crisco J.J. (2008). Head impact severity measures for evaluating mild traumatic brain injury risk exposure. Neurosurgery.

[bib19] Greer J.R., Deshpande V.S. (2019). Three-dimensional architected materials and structures: design, fabrication, and mechanical behavior. MRS Bull..

[bib20] Guo M., Pitet L.M., Wyss H.M., Vos M., Dankers P.Y., Meijer E. (2014). Tough stimuli-responsive supramolecular hydrogels with hydrogen-bonding network junctions. J. Am. Chem. Soc..

[bib21] Gurdjian E.S., Roberts V., Thomas L.M. (1966). Tolerance curves of acceleration and intracranial pressure and protective index in experimental head injury. J. Trauma Acute Care Surg..

[bib22] Han L., Liu K., Wang M., Wang K., Fang L., Chen H., Zhou J., Lu X. (2018). Mussel-inspired adhesive and conductive hydrogel with long-lasting moisture and extreme temperature tolerance. Adv. Funct. Mater..

[bib23] He Z., Wang F., Zhu Y., Wu H., Park H.S. (2017). Mechanical properties of copper octet-truss nanolattices. J. Mech. Phys. Sol..

[bib24] Hernández-Nava E., Smith C., Derguti F., Tammas-Williams S., Léonard F., Withers P., Todd I., Goodall R. (2016). The effect of defects on the mechanical response of Ti-6Al-4V cubic lattice structures fabricated by electron beam melting. Acta Materialia.

[bib25] Hu X., Vatankhah-Varnoosfaderani M., Zhou J., Li Q., Sheiko S.S. (2015). Weak hydrogen bonding enables hard, strong, tough, and elastic hydrogels. Adv. Mater..

[bib26] Jackson J.A., Messner M.C., Dudukovic N.A., Smith W.L., Bekker L., Moran B., Golobic A.M., Pascall A.J., Duoss E.B., Loh K.J. (2018). Field responsive mechanical metamaterials. Sci. Adv..

[bib27] Jacobsen A.J., Mahoney S., Carter W.B., Nutt S. (2011). Vitreous carbon micro-lattice structures. Carbon.

[bib28] Jiang Y., Wang Q. (2016). Highly-stretchable 3D-architected mechanical metamaterials. Sci. Rep..

[bib29] Khare E., Holten-Andersen N., Buehler M.J. (2021). Transition-metal coordinate bonds for bioinspired macromolecules with tunable mechanical properties. Nat. Rev. Mater..

[bib30] Koons G.L., Diba M., Mikos A.G. (2020). Materials design for bone-tissue engineering. Nat. Rev. Mater..

[bib31] Kudo A., Misseroni D., Wei Y., Bosi F. (2019). Compressive response of non-slender octet carbon microlattices. Front. Mater..

[bib32] Kumar R.M., Baskar P., Balamurugan K., Das S., Subramanian V. (2012). On the perturbation of the H-bonding interaction in ethylene glycol clusters upon hydration. J. Phys. Chem. A.

[bib33] Lee I.-N., Dobre O., Richards D., Ballestrem C., Curran J.M., Hunt J.A., Richardson S.M., Swift J., Wong L.S. (2018). Photoresponsive hydrogels with photoswitchable mechanical properties allow time-resolved analysis of cellular responses to matrix stiffening. ACS Appl. Mater. Interfaces.

[bib34] Lee J.H., Han W.J., Jang H.S., Choi H.J. (2019). Highly tough, biocompatible, and magneto-responsive Fe 3 O 4/laponite/PDMAAm nanocomposite hydrogels. Sci. Rep..

[bib35] Liang R., Yu H., Wang L., Lin L., Wang N., Naveed K.-u.-R. (2019). Highly tough hydrogels with the body temperature-responsive shape memory effect. ACS Appl. Mater. Interfaces.

[bib36] Lifson M.L. (2019). Electromechanical Properties of 3D Multifunctional Nano-Architected Materials.

[bib37] Lin P., Ma S., Wang X., Zhou F. (2015). Molecularly engineered dual-crosslinked hydrogel with ultrahigh mechanical strength, toughness, and good self-recovery. Adv. Mater..

[bib38] Liu J., Scherman O.A. (2018). Cucurbit [n] uril supramolecular hydrogel networks as tough and healable adhesives. Adv. Funct. Mater..

[bib39] Liu J., Tan C.S.Y., Yu Z., Lan Y., Abell C., Scherman O.A. (2017). Biomimetic supramolecular polymer networks exhibiting both toughness and self-recovery. Adv. Mater..

[bib40] Liu X.J., Li H.Q., Zhang B.Y., Wang Y.J., Ren X.Y., Guan S., Gao G.H. (2016). Highly stretchable and tough pH-sensitive hydrogels with reversible swelling and recoverable deformation. Rsc Adv..

[bib41] Lou D., Wang C., He Z., Sun X., Luo J., Li J. (2019). Robust organohydrogel with flexibility and conductivity across the freezing and boiling temperatures of water. Chem. Commun..

[bib42] Luo F., Sun T.L., Nakajima T., Kurokawa T., Zhao Y., Sato K., Ihsan A.B., Li X., Guo H., Gong J.P. (2015). Oppositely charged polyelectrolytes form tough, self-healing, and rebuildable hydrogels. Adv. Mater..

[bib43] Maskery I., Aboulkhair N., Aremu A., Tuck C., Ashcroft I., Wildman R.D., Hague R.J. (2016). A mechanical property evaluation of graded density Al-Si10-Mg lattice structures manufactured by selective laser melting. Mater. Sci. Eng. A.

[bib44] Mei H., Zhao R., Xia Y., Du J., Wang X., Cheng L. (2019). Ultrahigh strength printed ceramic lattices. J. Alloys Compounds.

[bib45] Mo F., Liang G., Meng Q., Liu Z., Li H., Fan J., Zhi C. (2019). A flexible rechargeable aqueous zinc manganese-dioxide battery working at− 20° C. Energy Environ. Sci..

[bib46] Morelle X.P., Illeperuma W.R., Tian K., Bai R., Suo Z., Vlassak J.J. (2018). Highly stretchable and tough hydrogels below water freezing temperature. Adv. Mater..

[bib47] O’Masta M.R., Dong L., St-Pierre L., Wadley H., Deshpande V.S. (2017). The fracture toughness of octet-truss lattices. J. Mech. Phys. Sol..

[bib48] Peng F., Li G., Liu X., Wu S., Tong Z. (2008). Redox-responsive gel− sol/sol− gel transition in poly (acrylic acid) aqueous solution containing Fe (III) ions switched by light. J. Am. Chem. Soc..

[bib49] Peng K., Yang K., Fan Y., Yasin A., Hao X., Yang H. (2017). Thermal/light dual-activated shape memory hydrogels composed of an agarose/poly (acrylamide-co-acrylic acid) interpenetrating network. Macromolecular Chem. Phys..

[bib50] Ren Y., Guo J., Liu Z., Sun Z., Wu Y., Liu L., Yan F. (2019). Ionic liquid–based click-ionogels. Sci. Adv..

[bib51] Rong Q., Lei W., Chen L., Yin Y., Zhou J., Liu M. (2017). Anti-freezing, conductive self-healing organohydrogels with stable strain-sensitivity at subzero temperatures. Angew. Chem. Int. Ed. Engl..

[bib52] Saleh M.S., Hu C., Panat R. (2017). Three-dimensional microarchitected materials and devices using nanoparticle assembly by pointwise spatial printing. Sci. Adv..

[bib53] Schaedler T.A., Carter W.B. (2016). Architected cellular materials. Annu. Rev. Mater. Res..

[bib54] Schaedler T.A., Jacobsen A.J., Torrents A., Sorensen A.E., Lian J., Greer J.R., Valdevit L., Carter W.B. (2011). Ultralight metallic microlattices. Science.

[bib55] Schaedler T.A., Ro C.J., Sorensen A.E., Eckel Z., Yang S.S., Carter W.B., Jacobsen A.J. (2014). Designing metallic microlattices for energy absorber applications. Adv. Eng. Mater..

[bib56] Schwaiger R., Meza L., Li X. (2019). The extreme mechanics of micro-and nanoarchitected materials. MRS Bull..

[bib57] Shao C., Chang H., Wang M., Xu F., Yang J. (2017). High-strength, tough, and self-healing nanocomposite physical hydrogels based on the synergistic effects of dynamic hydrogen bond and dual coordination bonds. ACS Appl. Mater. Interfaces.

[bib58] Sui X., Guo H., Chen P., Zhu Y., Wen C., Gao Y., Yang J., Zhang X., Zhang L. (2020). Zwitterionic osmolyte-based hydrogels with antifreezing property, high conductivity, and stable flexibility at subzero temperature. Adv. Funct. Mater..

[bib59] Sun J.-Y., Zhao X., Illeperuma W.R., Chaudhuri O., Oh K.H., Mooney D.J., Vlassak J.J., Suo Z. (2012). Highly stretchable and tough hydrogels. Nature.

[bib60] Sun T.L., Kurokawa T., Kuroda S., Ihsan A.B., Akasaki T., Sato K., Haque M.A., Nakajima T., Gong J.P. (2013). Physical hydrogels composed of polyampholytes demonstrate high toughness and viscoelasticity. Nat. Mater..

[bib61] Surjadi J.U., Feng X., Fan R., Lin W., Li X., Lu Y. (2021). Hollow medium-entropy alloy nanolattices with ultrahigh energy absorption and resilience. NPG Asia Mater..

[bib62] Surjadi J.U., Feng X., Zhou W., Lu Y. (2021). Optimizing film thickness to delay strut fracture in high-entropy alloy composite microlattices. Int. J. Extreme Manufacturing.

[bib63] Surjadi J.U., Gao L., Cao K., Fan R., Lu Y. (2018). Mechanical enhancement of core-shell microlattices through high-entropy alloy coating. Sci. Rep..

[bib64] Surjadi J.U., Gao L., Du H., Li X., Xiong X., Fang N.X., Lu Y. (2019). Mechanical metamaterials and their engineering applications. Adv. Eng. Mater..

[bib65] Tancogne-Dejean T., Spierings A.B., Mohr D. (2016). Additively-manufactured metallic micro-lattice materials for high specific energy absorption under static and dynamic loading. Acta Materialia.

[bib66] Tao R., Ji L., Li Y., Wan Z., Li T., Wu W., Liao B., Ma L., Fang D. (2020). 4D printed origami metamaterials with tunable compression twist behavior and stress-strain curves. Composites B Eng..

[bib67] Thiyagasundaram P., Sankar B.V., Arakere N.K. (2010). Elastic properties of open-cell foams with tetrakaidecahedral cells using finite element analysis. AIAA J..

[bib68] Tsopanos S., Mines R., McKown S., Shen Y., Cantwell W., Brooks W., Sutcliffe C. (2010). The influence of processing parameters on the mechanical properties of selectively laser melted stainless steel microlattice structures. J. Manufacturing Sci. Eng..

[bib69] Wang Y., Ramirez B., Carpenter K., Naify C., Hofmann D.C., Daraio C. (2019). Architected lattices with adaptive energy absorption. Extreme Mech. Lett..

[bib70] Warmuth F., Osmanlic F., Adler L., Lodes M.A., Körner C. (2016). Fabrication and characterisation of a fully auxetic 3D lattice structure via selective electron beam melting. Smart Mater. Structures.

[bib71] Xia S., Song S., Li Y., Gao G. (2019). Highly sensitive and wearable gel-based sensors with a dynamic physically cross-linked structure for strain-stimulus detection over a wide temperature range. J. Mater. Chem. C.

[bib72] Yan C., Hao L., Hussein A., Bubb S.L., Young P., Raymont D. (2014). Evaluation of light-weight AlSi10Mg periodic cellular lattice structures fabricated via direct metal laser sintering. J. Mater. Process. Technol..

[bib73] Yang C., Boorugu M., Dopp A., Ren J., Martin R., Han D., Choi W., Lee H. (2019). 4D printing reconfigurable, deployable and mechanically tunable metamaterials. Mater. Horizons.

[bib74] Yang C.H., Wang M.X., Haider H., Yang J.H., Sun J.-Y., Chen Y.M., Zhou J., Suo Z. (2013). Strengthening alginate/polyacrylamide hydrogels using various multivalent cations. ACS Appl. Mater. Interfaces.

[bib75] Zhang T., Silverstein M.S. (2017). Doubly-crosslinked, emulsion-templated hydrogels through reversible metal coordination. Polymer.

[bib76] Zhang W., Chen J., Li X., Lu Y. (2020). Liquid metal-polymer microlattice metamaterials with high fracture toughness and damage recoverability. Small.

[bib77] Zhang X., Wang Y., Ding B., Li X. (2020). Design, fabrication, and mechanics of 3D micro-/nanolattices. Small.

[bib78] Zheng S.Y., Ding H., Qian J., Yin J., Wu Z.L., Song Y., Zheng Q. (2016). Metal-coordination complexes mediated physical hydrogels with high toughness, stick–slip tearing behavior, and good processability. Macromolecules.

[bib79] Zheng X., Lee H., Weisgraber T.H., Shusteff M., DeOtte J., Duoss E.B., Kuntz J.D., Biener M.M., Ge Q., Jackson J.A. (2014). Ultralight, ultrastiff mechanical metamaterials. Science.

[bib80] Zheng X., Smith W., Jackson J., Moran B., Cui H., Chen D., Ye J., Fang N., Rodriguez N., Weisgraber T. (2016). Multiscale metallic metamaterials. Nat. Mater..

