# Supplementary material for: 3D architected temperature-tolerant organohydrogels with ultra-tunable energy absorption
Source: iScience. 2021 Jun 26;24(7):102789. doi: 10.1016/j.isci.2021.102789 (PMC8271157; doi:10.1016/j.isci.2021.102789)
Supplement: Document S1. Figures S1–S6 [file mmc1.pdf]

iScience, Volume *24*

Supplemental information

3D architected temperature-tolerant organohydrogels with ultra-tunable energy absorption

James Utama Surjadi, Yongsen Zhou, Tianyu Wang, Yong Yang, Ji-jung Kai, Yang Lu, and Zuankai Wang

S1. Generalized Mechanical Property Model

The mechanical properties of the 3D octet organohydrogel lattices are governed by various material-dependent and geometry-dependent factors, which can be generalized in **Figure S5**.

S1.1 Material-dependent factors (applies to both bulk and architected organohydrogels):

Concentration and ratio of monomers – in our co-polymer system of PAA and PAM, changing the molar concentration of the monomer with respect to the solvent, as well as the relative ratio of PAA to PAM would induce changes to the mechanical properties of the hydrogel/organohydrogel produced.(Lin et al., 2015)

Degree of crosslinking – the degree of crosslinking or polymerization could be controlled by varying the amount of crosslinker in the resin, and to a lesser degree, the irradiation time used for photopolymerization. Increasing the amount of crosslinking would result in higher modulus and strength, but at the expense of reduced deformability, which beyond a certain point will cause brittle fracture.

Type and concentration of cations – the type of cations used could influence the strength of metal-coordination bonds formed between the carboxyl group and the metal cations. The trivalent ionic interaction used in our work have been proven previously to be stronger than other monovalent or divalent interactions.(Zheng et al., 2016) As experimentally demonstrated in **Figure 3**, we have also optimized the concentration of the cations within the hydrogel/organohydrogel to achieve the best strength, stiffness, and toughness/energy absorption.

Ratio of solvents – the ratio of individual solvents within the binary solvent system could also influence the mechanical properties and temperature tolerance of the organohydrogel.(Chen et al., 2018; Rong et al., 2017) In our case, the effect is accentuated due to the presence of the metal-coordination bonds. For instance, partially displacing water with EG via solvent exchange results in a drastic improvement in both the mechanical properties and temperature tolerance of the metal-coordinated organohydrogel as opposed to pure hydrogel.

In our work, we primarily focus on quantifying the most significant and beneficial material-dependent parameters that would contribute to the ultra-large tunable range in mechanical properties, such as the influence on Fe^{3+} concentration and binary solvent on the strength, modulus, and energy absorption of the hydrogels and organohydrogels.

S1.2 Geometry-dependent factors (applies only to architected organohydrogels):

Type of architecture – the type of architecture employed plays a critical role in determining the mechanical properties and deformation behaviour of the architected organohydrogels.(Deshpande et al., 2001a; Fleck et al., 2010; Greer and Deshpande, 2019; Schaedler and Carter, 2016; Schwaiger et al., 2019) In this case, we chose the truss-based lattices which are known to be the most versatile and easy to manufacture across all length scales.(Zhang et al., 2020) For cellular truss-based lattices, their deformation behaviours could generally be classified into two main classes, namely bending-dominated and stretching-dominated behaviour.(Deshpande *et al.*, 2001a) Bending - dominated structures, such as conventional foams, typically deform by bending of its interconnecting members (such as struts and plates), whereas stretching - dominated cellular materials, which can be represented by a triangulated arrangement of such interconnecting members, deform through the uniaxial compression and/or tension of its elements. Maxwell's criterion is usually used to determine whether a cellular architecture is either bending- or stretching-dominated, although there are a few unusual exceptions. Stretching-dominated lattices, such as the octet-truss lattice employed in our work, possess enhanced load-bearing efficiency over bending-dominated lattices.(Deshpande et al., 2001b) This means that at the same mass, stretching-dominated lattices will exhibit higher modulus and strength compared to bending-dominated lattices.

Besides the difference in mechanical properties, the type of lattice used also influences the shape of the stress-strain curve obtained. Assuming that the material constituent is sufficiently deformable, bending-

dominated lattices typically exhibit stress-strain curves like conventional foams or bulk hydrogels/organohydrogels, whereas stretching-dominated lattices could exhibit a “post-yield softening” behaviour, which for certain applications are desirable, such as foams for impact protection.(Ashby, 2006) For impact scenarios where the contact area remains constant, bending-dominated lattices are desirable due to the continual and gradual increase in stress. However, for impact scenarios where the contact area increases as the time/deformation increases, such as those in protective helmets, stretching-dominated lattices (used in this work) that softens upon yielding is desirable to reduce the impact force imposed upon the person it is supposed to protect.(Clough et al., 2019) Therefore, the ability to manipulate the mechanical properties and deformation behaviour of energy absorbing materials can be highly desirable for various applications.

Relative density – unlike traditional bulk organohydrogels where the density of the material is determined solely by its material composition, architected organohydrogels can vary their densities by changing the volume fraction (commonly referred to as relative density) of the structure used. Changing their densities would consequently induce mechanical property changes that is also dependent on the type of architecture employed.(Schaedler and Carter, 2016) In addition, varying their relative densities would also affect their deformation mechanisms and characteristics. For example, reducing the diameter of the interconnected beams or struts (i.e. higher aspect ratio) or increasing the unit cell size would reduce the overall density of the metamaterial created. Furthermore, the relative density of the lattices plays a crucial role in determining both the position of yield or buckling strain and densification strain under compression (where the stress rises steeply) of the lattices' stress-strain curve (e.g. lowering the relative density results in higher densification strain, and vice versa).(Jiang and Wang, 2016; Warmuth et al., 2016) From an application perspective of impact attenuating helmets, higher densification strain could prolong the duration of impact, which results in lower transmitted force to the user's head.(Clough *et al.*, 2019)

Number of unit cells – Changing the number of unit cells could influence the stress-strain response of a lattice metamaterial. For example, increasing the number of unit cells along the loading direction could change the number and magnitude of “post-yield softening” regions on the compression stress-strain curve of a stretching-dominated octet lattice metamaterial of similar size.(Jiang and Wang, 2016)

Spatial arrangement/distribution – the arrangement and spatial distribution of unit cells within a lattice metamaterial could significantly influence both the overall mechanical properties and deformation behaviour of the metamaterial. For instance, designing a bi-phase lattice with one phase composed of lattices with low relative density and the other phase with high relative density could strengthening of the overall lattice metamaterial once a certain strain limit is reached (i.e. once all the layers composed of the lower relative density unit cells have yielded/buckled).(Greer and Deshpande, 2019; Lifson, 2019) In addition, it is also possible to fabricate functionally graded lattices that has a gradual variation in relative density (i.e. beams/struts with increasing thickness) along the loading direction to provide a more gradual and controlled deformation behaviour. This strategy has been demonstrated to potentially enhance the fracture toughness of lattices as well.(Maskery et al., 2016)

To summarize, the tunable mechanical properties of our organohydrogels comes from the varying concentration of Fe^{3+} within the organohydrogel. The influence of Fe^{3+} concentration is then combined with the influence from other material-dependent and geometry-dependent factors to give the overall mechanical properties and deformation behaviour of the architected organohydrogel. In general, the mechanical properties of the architected organohydrogels could be summarized through the following expression:

$$P = (M_{MC} M_{CD} M_{CC} M_{RS}) \times (G_{TA} G_{RD} G_{NC} G_{SD}) \qquad (equation\ 1)$$

where P represents a specific mechanical property (e.g. stiffness, strength, energy absorption), all the M_{xx} terms represent the influence of each material-dependent parameters, and G_{xx} terms represent the influence of each geometry-dependent parameters. In more details, M_{MC}, M_{CD}, M_{CC}, M_{RS} are the mechanical contributions of the relative monomer concentrations with respect to the solvent(s), crosslinking density, cation concentration, and relative ratio of solvents in the binary solvent, respectively. G_{TA}, G_{RD}, G_{NC}, G_{SD} are the mechanical contributions of type of architecture, relative density, number of unit cells, and 3D spatial distribution, respectively. As M_{CC}, which is related to the concentration of Fe^{3+}, is the only tunable

parameter upon manufacture due to the stimuli-responsive nature of metal coordination bonds, we kept the influence from all the other parameters constant in our work.

In addition, to provide a clearer picture on how the dynamically and topologically controlled properties of these architected organohydrogels could be linked to end use applications, we used a simple example of how the mechanical properties and deformation behaviour of an organohydrogel lattice can be tailored to create efficient impact attenuators for protective sports helmet (**Figure S6**).

S2. Mechanical Property Analysis

The relationship between the 3D architected structure with respect to the intrinsic material properties can be summarized as follows:

Relative density

The relative density, $\rho_{relative}$, of the octet-truss lattice material (ratio of the density of the lattice material to the density of the solid material from which it is made) can be obtained through a CAD software (e.g. SolidWorks) by obtaining the volume fraction of the lattice with respect to a solid cube, or can be approximated analytically by the following equation:(O'Masta et al., 2017)

$$\rho_{relative} = 6\sqrt{2}\left(\frac{d}{L}\right)^2 \qquad (equation\ 2)$$

where d represents the diameter of the strut, and L represents the length of each cubic unit cell.

Stiffness

The effective stiffness of the octet lattice was first investigated by assuming that the octet lattice is a stretching dominated solid with pin-jointed struts with negligible bending effects.(Deshpande *et al.*, 2001b) This was later verified by other researchers to be true for a relative density of lower than 0.1 and strut slenderness *r/l* lower than 0.06.(Dong et al., 2015; He et al., 2017) From the analysis that the octet unit cell free to deform laterally due to Poisson effect, the lattice stiffness can be given as:

$$E_{effective} = \frac{2\pi\sqrt{2}}{3} E\left(\frac{r}{l}\right)^2 K_b \qquad (equation\ 3)$$

where E is the stiffness or Young's modulus of the constituent material, and K_b is a coefficient that accounts for bending effects. For truss architectures with pin-joined struts, K_b = 1, while frame lattices that show non-negligible bending effects have nodal rigidity K_b > 1, and K_b can be written as follows:(Kudo et al., 2019)

$$K_b = \frac{1 + 15\left(\frac{r}{l}\right)^2 + 36\left(\frac{r}{l}\right)^4}{1 + 7\left(\frac{r}{l}\right)^2} \qquad (equation\ 4)$$

Equations 2 and 3 are obtained by means of the classical beam theory which predicts that the effective Poisson's ratio, *v*, of the lattice is independent of the relative density and is equal to 0.33. However, numerical simulations have proven that both bending-dominated(Thiyagasundaram et al., 2010) and stretching-dominated(Tancogne-Dejean et al., 2016) lattices experience a decrease of *v* with an increase of relative density. Lattices with high relative density could show limited lateral expansion when compressed. Similarly, the lattices studied in this work presented a relative density greater than 0.1 and non-slender struts. These features contribute to limit the lateral displacement of the microlattices, which experimentally showed a negligible effective Poisson's ratio. Therefore, a more accurate expression of the effective Young's modulus of the non-slender cubic octet lattices can be obtained by the following:(Kudo *et al.*, 2019)

$$E_{effective} = \sqrt{2}\pi E\left(\frac{r}{l}\right)^2\left(1+3\left(\frac{r}{l}\right)^2\right) \qquad (equation\ 5)$$

Strength

The compressive strength of an octet-truss lattice material was determined considering the two possible failure mechanisms, namely elastic buckling, or plastic yielding. If the lattice has a low relative density, elastic buckling will be responsible of the collapse of the octet-truss lattice. However, when the strut diameter and relative density of the lattice increases, buckling is preceded by strut yielding as the stress reaches the material yield strength, σ_m. Generally, the compressive strength, σ_l, for an octet lattice can be expressed as:(Deshpande *et al.*, 2001b)

$$\sigma_l = \min\left\{\frac{\sqrt{2}\pi^3 n^2 E}{2}\left(\frac{r}{l}\right)^4 ; 2\sqrt{2}\pi\left(\frac{r}{l}\right)^2 \sigma_m\right\} \qquad (equation\ 6)$$

Like the analytical evaluation of stiffness, equation 5 could also be extended to account for non-slender lattices with laterally constrained unit cells. A more accurate representation of the cubic octet lattices presented in this work could be expressed by the following:(Kudo *et al.*, 2019)

$$\sigma_l = \min\left\{\frac{\sqrt{2}\pi^3 n^2 E}{2}\left(\frac{r}{l}\right)^4\left(1+3\left(\frac{r}{l}\right)^2\right); 2\sqrt{2}\pi\left(\frac{r}{l}\right)^2\frac{\left(1+3\left(\frac{r}{l}\right)^2\right)}{\left(1+6\left(\frac{r}{l}\right)^2\right)}\sigma_m\right\} \qquad (equation\ 7)$$

Predicting stress-strain curve

Due to the effects of various geometrical parameters such as number of unit cells and material-property dependent stress-strain response, it is challenging to accurately fit a general analytical equation for the stress-strain curve of the octet organohydrogel lattice under compression with different concentrations of Fe^{3+}. However, it is possible to obtain representative polynomial fitting curves to characterize the stress-strain response of architected organohydrogels at each Fe^{3+} concentration. For example, the stress-strain curve of the 1M OHG-Fe^{3+} lattices can be approximated by the following 5th order polynomial equation:

$$y = 0.03743 + 136.8x^1 - 488.6x^2 - 845.6x^3 + 5966.6x^4 - 6479.4x^5 \quad (equation\ 8)$$

where y in this case represents the compressive stress in MPa and x represents the compressive strain the architected organohydrogel is subjected to.

Energy absorption

The energy absorption per unit volume, W, of the octet lattice can then be evaluated as the area under the stress-strain curve, given by:(Schaedler et al., 2014; Tancogne-Dejean *et al.*, 2016)

$$W = \int_0^{\varepsilon_D} \sigma d\varepsilon \qquad (equation\ 9)$$

where ε_D represents the densification strain and σ is the compressive stress.

Specific stiffness, strength, and energy absorption

After obtaining the absolute stiffness ($E_{effective}$), absolute strength (σ_l), and energy absorption per unit volume (W) for each organohydrogel lattices, the corresponding specific stiffness ($E_{specific}$), strength

($\sigma_{specific}$), and energy absorption ($W_{specific}$) could be calculated by dividing the values by the lattice density, $\rho_{lattice}$, which can be given as:

$$E_{specific} = \frac{E_{effective}}{\rho_{lattice}} \quad (equation\ 10)$$

$$\sigma_{specific} = \frac{\sigma_l}{\rho_{lattice}} \quad (equation\ 11)$$

$$W_{specific} = \frac{W}{\rho_{lattice}} \quad (equation\ 12)$$

$$\rho_{lattice} = \rho_{relative} \times \rho_{bulk} \quad (equation\ 13)$$

where ρ_{bulk} represents the bulk density of the organohydrogel material.

Nevertheless, it is important to note that all these equations can be further improved to account for the manufacturing-induced imperfections such as non-cubic unit cells, orientation-dependent beam slenderness, and increased nodal volume, whose effects are highly variable depending on the manufacturing technique employed, making it challenging to fully them characterized solely by analytical methods.

Supplemental Figures

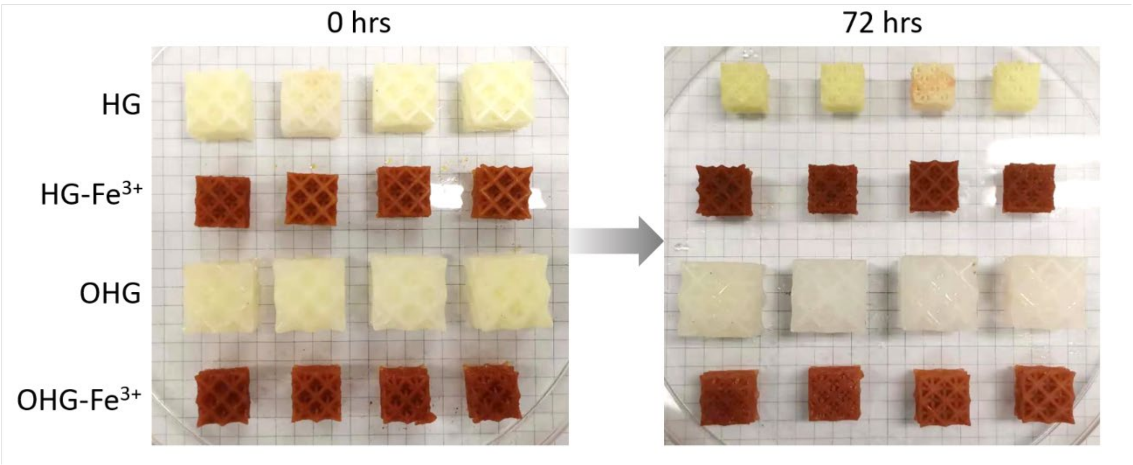

Figure S1. Non-drying properties of the architected organohydrogels compared to hydrogels. Related to Figure 2. Photographs of the hydrogel and organohydrogel lattices after leaving it in ambient environment for 0 hours and 72 hours, showing that the organohydrogel lattices show little to no difference in size and mass while the hydrogel lattices all shrunk and became brittle after 72 hours.

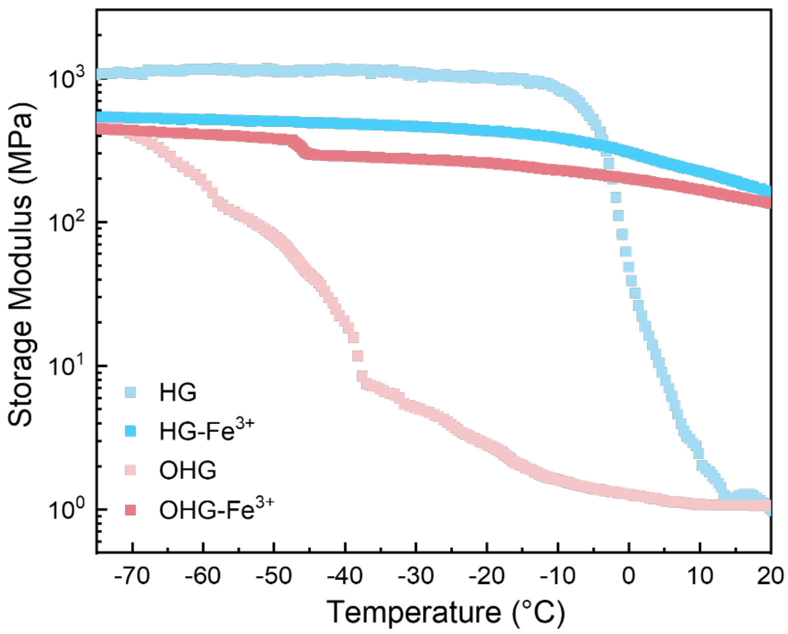

Figure S2. DMA measurement of the fabricated hydrogel and organohydrogel lattices. Related to Figure 2.

(a)

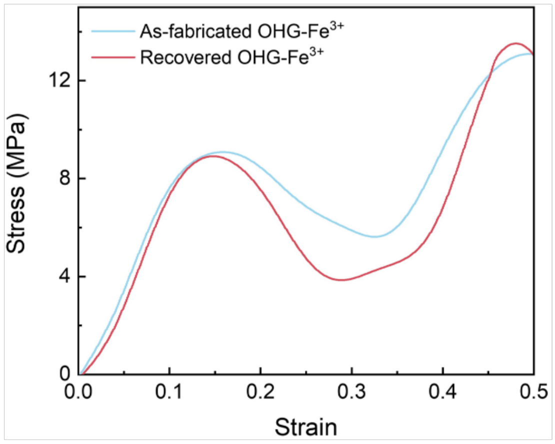

(b)

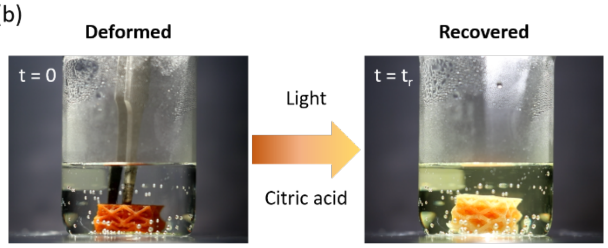

Light intensity, I_0 (W/cm^2)	Citric acid concentration (M)	Time taken for recovery, t_r (s)
0.002	0.10	288 ± 20
0.002	0.25	280 ± 20
0.002	0.50	276 ± 20
0.1	0.25	60 ± 5
0.2	0.25	30 ± 5

Figure S3. Summary of the deconstruction and reconstruction of the metal coordination bonds. Related to Figure 3. (a) Stress-strain curves of the dual-crosslinked architected organohydrogels using as-fabricated and recovered lattices. (b) Influence of citric acid concentration and light intensity on the time taken for the deformed lattices to recover its original shape at 90℃.

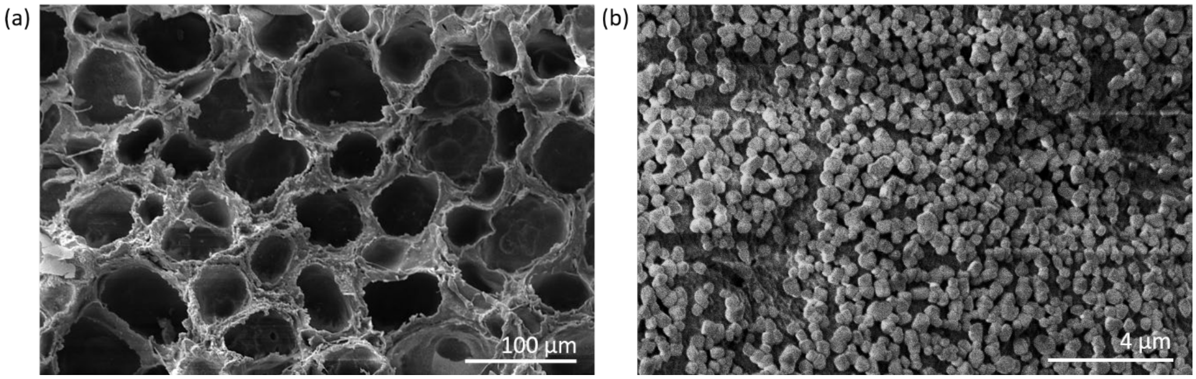

Figure S4. Microstructural evolution in metal-coordinated hydrogels. Related to Figure 3. Scanning Electron Microscopic (SEM) images of the (a) hydrogel (HG) and (b) metal-coordinated hydrogel (HG-Fe^{3+}), showing the presence of iron salt particles and increased density of polymer chains.

Traditional metal-coordinated PAA/PAM organohydrogel	Architected metal-coordinated PAA/PAM organohydrogel
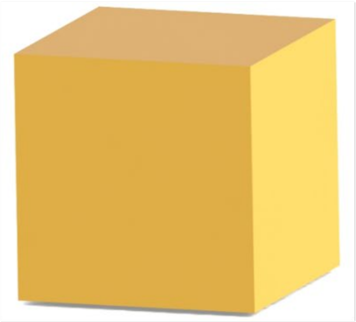	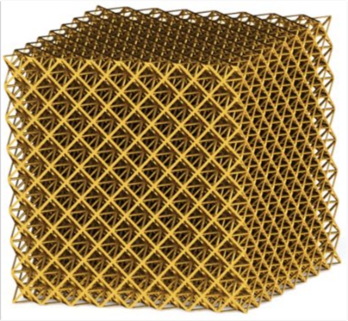
• Ratio of monomers<br>• Degree of crosslinking<br>• Concentration of cations<br>• Ratio of solvents	• Ratio of monomers<br>• Degree of crosslinking<br>• Concentration of cations<br>• Ratio of solvent<br>• Type of architecture<br>• Relative density<br>• Number of unit cells<br>• Spatial arrangement/distribution

Figure S5. Factors influencing the mechanical properties of traditional metal-coordinated bulk organohydrogels and architected metal-coordinated organohydrogels. Related to Figure 5.

Desired mechanical properties and response to an impact scenario:

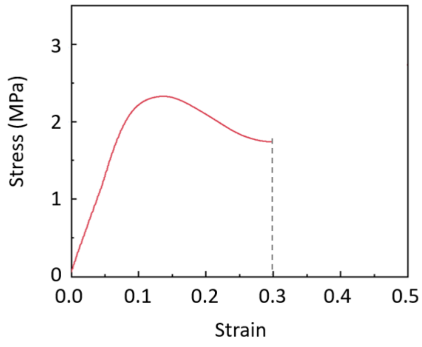

	Material-dependent parameters	Geometry-dependent parameters
How to obtain desired yield strength?	$M_{MC}, M_{CD}, M_{CC}, M_{RS}$	G_{TA}, G_{RD}, G_{SD}
How to obtain desired shape of stress-strain curve?	n/a	G_{TA}, G_{NC}, G_{SD}
How to obtain desired yield and densification strain?	n/a	G_{RD}

Figure S6. Example on how the properties of an architected organohydrogel could be tailored for a specific application. Related to Figure 5. M_{MC}, M_{CD}, M_{CC}, M_{RS} are the mechanical contributions of the relative monomer concentrations with respect to the solvent(s), crosslinking density, cation concentration, and relative ratio of solvents in the binary solvent, respectively. G_{TA}, G_{RD}, G_{NC}, G_{SD} are the mechanical contributions of type of architecture, relative density, number of unit cells, and 3D spatial distribution, respectively.
